# Induction of somatic mutations by low concentrations of tritiated water (HTO): evidence for the possible existence of a dose-rate threshold

**DOI:** 10.1093/jrr/rrab022

**Published:** 2021-04-24

**Authors:** Haruki Nagashima, Yuki Hayashi, Yuki Sakamoto, Kenshi Komatsu, Hiroshi Tauchi

**Affiliations:** 1 Department of Biological Sciences, Faculty of Science, Ibaraki University, Bunkyo 2-1-1, Mito, Ibaraki 310-8512 Japan; 2 Department of Genome Repair Dynamics, Radiation Biology Center, Kyoto University, Yoshida-Konoe Cho, Sakyo-ku, Kyoto 606-8501, Japan

**Keywords:** mutations, low dose, tritium

## Abstract

Tritium is a low energy beta emitter and is discharged into the aquatic environment primarily in the form of tritiated water (HTO) from nuclear power plants or from nuclear fuel reprocessing plants. Although the biological effects of HTO exposures at significant doses or dose rates have been extensively studied, there are few reports concerning the biological effects of HTO exposures at very low dose rates. In the present study using a hyper-sensitive assay system, we investigated the dose rate effect of HTO on the induction of mutations. Confluent cell populations were exposed to HTO for a total dose of 0.2 Gy at dose rates between 4.9 mGy/day and 192 mGy/day by incubating cells in medium containing HTO. HTO-induced mutant frequencies and mutation spectra were then investigated. A significant inflection point for both the mutant frequency and mutation spectra was found between 11 mGy/day and 21.6 mGy/day. Mutation spectra analysis revealed that a mechanistic change in the nature of the mutation events occurred around 11 mGy/day. The present observations and published experimental results from oral administrations of HTO to mice suggest that a threshold dose-rate for HTO exposures might exist between 11 mGy/day and 21.6 mGy/day where the nature of the mutation events induced by HTO becomes similar to those seen in spontaneous events.

## INTRODUCTION

Tritium is a radionuclide which is discharged into the aquatic environment, primarily in the form of tritiated water (HTO), by nuclear power plants and nuclear fuel reprocessing plants. In Japan, concerns about accumulating contaminated water containing radioisotopes grew after the Fukushima Dai-ichi Nuclear Power Plant (Fukushima Dai-ichi NPP) accident. Although the majority of radioisotopes in the contaminated water at the Fukushima Dai-ichi NPP can be removed by the Multi-nuclide Removal Facility or Advanced Liquid Processing System (ALPS), tritium (HTO) still remains in the water. Discharges of water which contain HTO have become a public issue due to concerns about the environmental and human health effects of tritium.

The biological effects of radiation are classified into two types: deterministic effects and stochastic effects. The difference between them is whether or not a dose threshold exists. A dose threshold for radiation can be seen for deterministic effects but cannot be seen for stochastic effects. Stochastic effects such as carcinogenesis are thought to be caused by genetic alterations such as somatic mutations. As the DNA damage yield increases in a dose dependent manner, the ‘linear non-threshold’ (LNT) model is considered to be an appropriate model to use to estimate the incidence of stochastic effects [1]. In this case, health risks would be expected to appear, even when the exposure dose or dose-rate is quite low. Stochastic effects may occur spontaneously, and the spontaneous frequency itself exhibits a wide scatter. In addition, the resulting effects are the same whether or not one is exposed to radiation. Therefore, it is difficult to distinguish radiation-induced events from spontaneous events, especially in the case of low-dose or low-dose-rate radiation exposures.

Hyper-sensitive systems including animal models and cellular models have been developed to study the effects of low-dose or low-dose-rate radiation [2–5]. To analyze the mutagenic effects of low-dose or low-dose-rate radiation, we previously developed a hypersensitive system which detects hypoxanthine-guanine phosphoribosyltransferase-deficient somatic mutations (Hprt). This cultured cell-based system uses an Hprt-deficient hamster cell line carrying a normal human X-chromosome [2]. The human *HPRT1* gene, which is located on the human X-chromosome, is used as a target for somatic mutations. In this cell line, any mutation event occurring on the human X-chromosome will not be associated with cell viability, thus, mutations can occur at a higher frequency than in a conventional system. Consequently, if DNA damage on the human X-chromosome is mis-repaired, the mis-repaired site would have no effect on cell viability, which allows the mis-repaired or altered site to persist in the cell. Thus, the mutation frequency detected in this cell system is about 50-fold higher than with a conventional cell system [2, 6].

In a previous study, mutant frequencies induced by low-dose X-rays were statistically significant at doses over 0.15 Gy when compared with the spontaneous mutant frequency, and a linear dose relationship with the mutant frequency was observed [7]. It was also found that the cultured cell system may permit us to distinguish radiation-induced events from spontaneous events by examining the mutation spectrum. There could be a critical transition level, where radiation-type events become significant at doses around 0.15 Gy [7].

Using the hypersensitive system, we previously investigated the effect of the dose rate on the appearance of HPRT-deficient mutations after tritium beta-ray exposures at dose rates above 21 mGy/day and found that a dose rate dependence was not observed at these dose rates regardless of the total dose (0.2 Gy, 0.3 Gy, 1 Gy) [2]. In contrast, there are some reports suggesting the existence of a ‘dose-rate threshold’ for HTO exposure [8, 9]. Because further studies at dose-rates less than 10 mGy/day were required, in the present study, we investigated the dose-rate dependency of mutant frequencies and mutation spectra after exposures to low concentrations of HTO in a confluent (G0/G1) population. Confluent cells were incubated in medium containing relatively low levels of HTO until the total dose reached 0.2 Gy. The mutant frequency and mutation spectra were then analyzed to determine if the major mutation events which occurred under culture conditions in the presence of a low concentration of HTO were radiation-type mutations. We found a significant inflection point for both, the mutant frequency and the mutation spectra, and there appeared to be a dose threshold, at dose-rates between 11 mGy/day and 21.6 mGy/day.

## MATERIALS AND METHODS

### Cells culture and irradiation

GM06318–10 cells were used in this study [6]. The GM06318–10 cell line is a subcloned hamster cell line that carries a human X-chromosome and is hyper-sensitive for mutation induction. Cells were cultured in D-MEM medium (Thermo-Fischer) supplemented with 5% fetal bovine serum (HyClone), 1 × hypoxanthine, aminopterin, thymidine (HAT) supplement (Thermo-Fischer), and 25 μg/ml gentamycin sulfate (SIGMA) and grown in a humidified 5% CO_2_/95% air atmosphere. For tritium beta-ray exposures, confluent (mainly G0/G1 phase) cells were incubated with a CO_2_-independent culture medium (Thermo-Fischer, supplemented with 0.2% FBS) containing an appropriate concentration of HTO. Cell cycle distributions of the confluent population were analyzed with an image cytometer (Tali cell analyzer, Thermo-Fischer), and the results showed that G0/G1-phase cells comprised about 94%, S-phase comprised about 5% and G2/M-phase cells comprised about 0.7% of the population. The HTO dose-rate was calculated according to a formula described elsewhere [10]. Cells were cultured for an appropriate period (from 25 h to 980 h) until the total dose reached 0.2 Gy. For low dose-rates which required exposure periods over 148 h, the HTO containing medium was exchanged for fresh medium every four to six days. The dose-rate and the concentration of HTO were as follows: 4.9 mGy/day (78 kBq/ml), 6.24 mGy/day (99 kBq/ml), 8.64 mGy/day (137 kBq/ml). 11.0 mGy/day (174 kBq/ml), 21.6 mGy/day (342 kBq/ml), 34.6 mGy/day (548 kBq/ml) and 192 mGy/day (3.04 MBq/ml). The total number of cells per flask at completion of the HTO exposure was 1.37 ± 0.41 × 10^7^ for control groups and 1.26 ± 0.37 × 10^7^ for exposed groups.

### Mutation assay

HPRT-deficient mutation assays using GM06318–10 cells were performed as described previously [2, 6, 7]. Briefly, on the last day of exposure, the HTO medium was changed to fresh medium without HTO and the cells were incubated for 30 min. The cells were then washed at least five time with fresh medium in order to remove HTO completely. The surviving fraction was measured using a portion of the cells, and the rest of the cells (more than 1 × 10^6^ cells) were distributed into eight dishes. After nine days of culture to permit expression of a mutant phenotype, the cells on each dish were trypsinized and inoculated into medium containing 5 μg/ml of 6-thioguanine (6-TG, Wako) at a density of 1 × 10^4^ cells per 100 mm dish. The cells were fixed after 14 days with ethanol and stained with a Giemsa solution (Merck). Because the spontaneous mutant frequency varied between experimental groups (the mean and SD was 54.5 ± 48.3 per 10^4^ cells), control groups were provided for each dose-rate group. The induced mutant frequency was calculated from the number of 6-TG-resistant colonies as previously described [2].

### Analysis of mutation spectra

A single independent colony was subcloned from each 6-TG-cultured dish. Each mutant clone was expanded and total genomic DNA was extracted. The DXS markers on the human X-chromosome were identified using polymerase chain reaction (PCR) as previously described [2, 6, 7]. Briefly, the primer sets used in this study were DXS86 (5’-CAATATTTACCTCCTCTGACAC-3′, 5′-ATGTTGAAAATGAAGATAAGGA-3′), DXS1194 (5’-CACCTCTGCCTTCCTCTCTATG-3′, 5’-TGGAAAAGGAACAATCAGAGTG-3′), DXS1048 (5’-TGGGTGTACATTGTGACTTT-TA-3′, 5’-TAAAATGTTGAGATGGACTTTG-3′), DXS1465 (5’-GCAATCAACCAAGAT- GGTTAC-3′, 5′- AACCTCTATAAAAGCAGGAAAATG −3′), and DXS1497 (5′- CTTG- ATAGGGGAAGATAAGG-3′, 5′- GAATGGTAGAGAGGAAGTTG-3′). Genomic DNA (~250 ng) was added to the mixture (15 μL) containing 1 unit of ExTaq polymerase (TaKaRa), 0.2 mM dNTPs, and the reaction buffer supplied with the polymerase. For DXS86, DXA1048 and DXS1194, the reaction was performed by heating to 95°C for 2 min and 30 cycles of DNA denaturation (95°C, 40 sec), annealing (56°C, 30 sec) and DNA polymerization (72°C, 1 min); for DXS1465, the parameters used were DNA denaturation (95°C, 40 sec), annealing (60°C, 30 sec), DNA polymerization (72°C, 40 sec); and for DXS1497 conditions were DNA denaturation (95°C, 40 sec), annealing (55°C, 30 sec), DNA polymerization (72°C, 40 sec). The PCR products were analyzed with 1% agarose gel electrophoresis.

### Statistical analysis

Experimental data obtained from at least three independent experiments were used for statistical analysis. Each data point is represented as the mean ± SD. Statistical analysis was performed with the multiple comparison method (Tukey–Kramer method), Fisher’s exact test, Chi-square test, or with linear regression analysis by using R software (R Project for Statistical Computing), and a value of p < 0.05 was considered statistically significant.

## RESULTS

### Induction of HPRT-deficient mutations after low-dose rate exposures to HTO

**Fig. 1.**
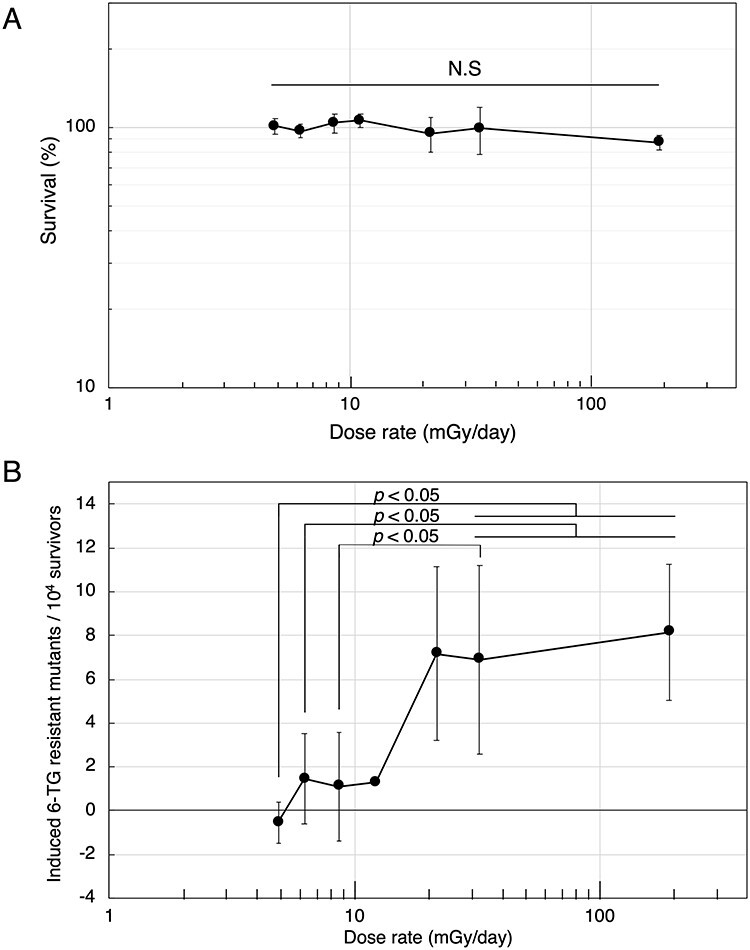
(A) Clonogenic survival following HTO exposures at various dose-rates. (B) Dose-rate dependency of the induced mutant frequency on HTO levels. Each data point represents the mean ± SD obtained from at least three independent experiments. The statistical significance was analyzed with the Tukey–Kramer method.

In the previous studies, we found that a dose rate dependence was not observed at dose rates above 21 mGy/day regardless of the total dose (0.2 Gy, 0.3 Gy, 1 Gy) [2]. After considering the dose rate effects reported by Yamamoto *et al.* [8, 9] suggesting the existence of a dose-rate threshold around 12 mGy/day, we decided to investigate whether or not a threshold dose rate could be found. Confluent (G0/G1) cells were exposed to HTO at dose rates from 4.9 mGy/day to 192 mGy/day. At the tested dose (0.2 Gy) and dose rates, cell viabilities were not significantly affected (Fig. 1A). Figure 1B shows the induced mutant frequencies obtained from at least three independent experiments. A significant decrease in mutant frequencies was observed when cells were exposed at dose rates of less than 11.0 mGy/day. The observed mutant frequencies for dose rates of less than 11.0 mGy/day were very similar to those observed for spontaneous mutations in the controls.

### Analysis of mutation spectra

**Fig. 2.**
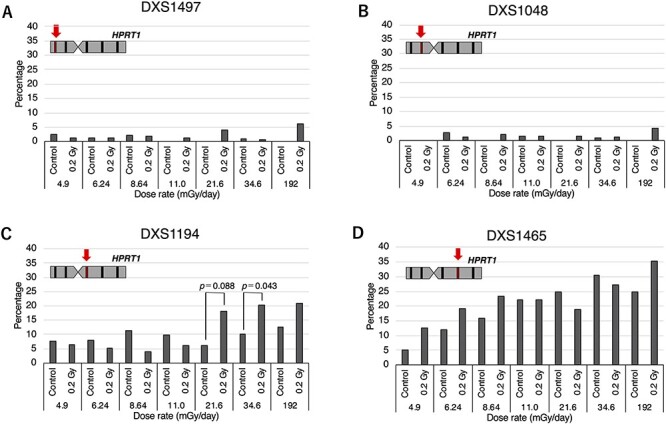
Percentage of deletions of STS markers on the human X-chromosome in the ‘non-spontaneous-type’ 6-TG resistant mutants induced by HTO exposures. The deletion rate of STS markers in the clones excluding the all negative (−/−/−/−) type clones are shown. (A) DXS1497 (Xp22.31) deletion. (B) DXS1048 (Xp11.22) deletion. (C) DXS1194 (Xq11.12) deletion. (D) DXS1465 (Xq21.33) deletion. The p-vales obtained by a statistical analysis with Fisher’s exact test are shown.

**Fig. 3.**
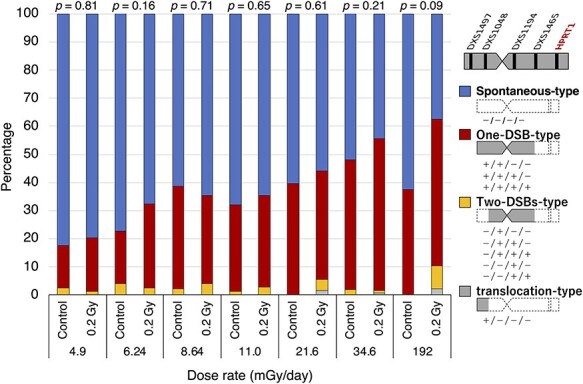
Mutation spectrum of 6-TG resistant mutants induced by HTO exposures. The mutation spectrum is classified by the existence or absence of the DXS1497/DXS1048/DXS1194/DXS1465 markers. The symbol ‘+’ indicates existence of the STS marker and the symbol ‘−’ indicates an absence of the STS marker. Mutants were classified into four categories on the basis of the minimum number of DSBs which would be required to generate the mutant: Spontaneous-type (−/−/−/−), One-DSB-type, Two-DSB-type and translocation-type. The number of clones analyzed at each dose-rate are shown in Table 1. The p-values for spontaneous-type vs radiation-type (one-DSB, two-DSBs and translocation) mutants between the control group and the 0.2 Gy group which were analyzed with Chi-square test are shown

**Fig. 4.**
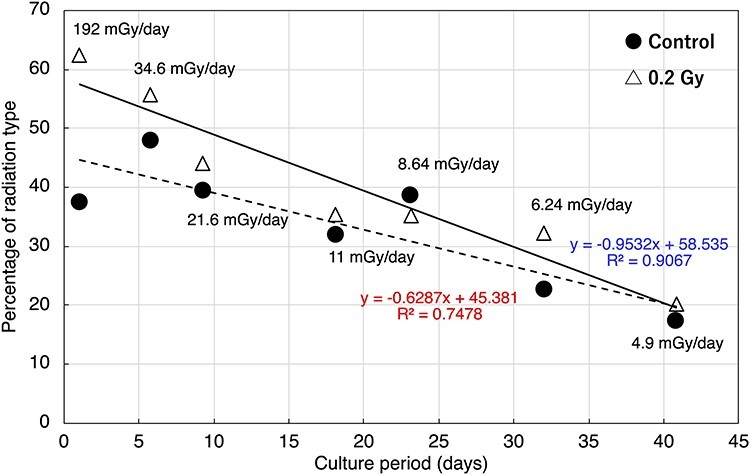
Culture period dependence of the percentage of radiation-type mutants. Mutations which could be generated by one DSB, two DSBs, or by more complicated damage (translocation) are defined as ‘radiation type.’ The solid line shows a fitted line of the 0.2 Gy irradiated group using the least squares method. The dotted line shows a fitted line for the spontaneous control group. Result from linear regression analysis are shown on the graph as a formula and correlation coefficient (R^2^) value.

The mutation spectra of HPRT-deficient (6-TG resistant) mutants induced by exposure to HTO were analyzed with PCR for sequence tagged site (STS) markers on the human X-chromosome. Tested STS markers were, DXS1497 (Xp22.31), DXS1048 (Xp11.22), DXS1194 (Xq11.12) and DXS1465 (Xq21.33). In the previous study, we found that more than 95% of spontaneously induced 6-TG resistant mutants had lost the DXS86 (Xq26) locus which is adjacent to the *HPRT1* gene [7]. Therefore, we performed PCR analysis for those four STS markers, and analysis for DXS86 was then performed only when the mutants contained STS markers on the long arm of human X-chromosome. The analysis showed that all the mutants, which retained the long arm of the human X-chromosome, lost the DXS86 locus. Thus, the approximate deletion size was estimated by a combination of these four STS makers which were present. More than 50% of the mutants were an ‘all negative’ (−/−/−/−) type and the percentage of the all negative type increased as the dose rate decreased (Table 1). Because the all negative type mutants could result from the loss of the human X-chromosome due to the instability of the human chromosome in rodent cells [11–13], this type of mutation was classified as a spontaneous-type mutation. In the case of a 4.9 mGy/day exposure, in which the culture period required to reach 0.2 Gy was over 40 days, the percentage of spontaneous-type mutations increased significantly in both, HTO exposed cells and in untreated control cells (p < 0.01 for 4.9 mGy/day groups vs 34.6 mGy/day groups by Chi-square test). This observation suggests the possibility that the rate of loss of the human X-chromosome is elevated with an increasing length of the culture period in non-selective (without HAT) medium. Figure 2 shows the frequency of loss of DXS markers in non-spontaneous-type mutants (mutant clones excluding the spontaneous-type deletion). In the non-spontaneous mutant clones, the percentage loss of DXS1497 and DXS1048, which are located on the short arm of the human X-chromosome, was negligible, regardless of whether the cells were irradiated or not, although a slight increase was seen in the group irradiated at 192 mGy/day (Figs 2A and B). The percentage loss of DXS1194 did not change with dose rates from 4.9 mGy/day to 11.0 mGy/day, however, it was clearly elevated in cells irradiated at or above 21.6 mGy/day (Fig. 2C). The percentage loss of DXS1465 tended to increase depending on both, the culture period and the dose rate (Fig. 2D). Based on the minimum number of DNA double-strand breaks (DSBs) required to form the mutants with each STS marker combination, we classified mutation types into four categories (Fig. 3): the one-DSB type represents a deletion on the long arm containing the *HPRT1* gene; the two-DSBs type represents deletions on both, the long and short arm; the translocation type represents loss of a centromere; and spontaneous-type deletions. The trend for the mutation spectra in groups irradiated with lower dose rates became similar to those seen in the controls. In contrast, at higher dose-rates, especially at 192 mGy/day, the percentage of spontaneous-type mutations markedly decreased and the percentage of translocation type mutations was increased (Fig. 3). When one-DSB type, two-DSB type and translocation types are classified as radiation type mutations, the percentage of radiation type mutations decreased depending on the length of the culture period (Fig. 4). Because the slope for HTO-exposed groups is steeper than control groups, the percentage of radiation-type mutations approached the values seen in control groups (Fig. 4).

**Table 1:** Mutation spectrum analysis of 6-thioguanine-resistant mutants induced by HTO exposure.

Dose rate (mGy/day)		Number of mutant clones analyzed	DXS1497 (Xp22.31)/DXS1048 (Xp11.22)/DXS1194 (Xq11.12)/DXS1465 (Xq21.33)									
			−/−/−/−	+/+/−/−	+/+/+/−	+/+/+/+	−/+/−/−	−/+/+/−	−/+/+/+	−/−/+/−	−/−/+/+	+/−/−/−
			Number of clones (%)	Number of clones (%)	Number of clones (%)	Number of clones (%)	Number of clones (%)	Number of clones (%)	Number of clones (%)	Number of clones (%)	Number of clones (%)	Number of clones (%)
4.9	Control	40	33 (82.5)	3 (7.5)	1 (2.5)	2 (5.0)	0 (0)	1 (2.5)	0 (0)	0 (0)	0 (0)	0 (0)
	0.2 Gy	79	63 (79.7)	5 (6.3)	9 (11.4)	1 (1.3)	0 (0)	1 (1.3)	0 (0)	0 (0)	0 (0)	0 (0)
6.24	Control	75	59 (78.7)	5 (6.7)	8 (10.7)	1 (1.3)	1 (1.3)	0 (0)	0 (0)	1 (1.3)	1 (1.3)	0 (0)
	0.2 Gy	152	103 (67.8)	7 (4.6)	26 (17.1)	12 (7.9)	1 (0.7)	1 (0.7)	0 (0)	2 (1.3)	0 (0)	0 (0)
8.64	Control	44	27 (61.4)	5 (11.4)	7 (15.9)	4 (9.1)	0 (0)	0 (0)	1 (2.3)	0 (0)	0 (0)	0 (0)
	0.2 Gy	99	64 (64.6)	4 (4.0)	19 (19.2)	8 (8.1)	0 (0)	2 (2.0)	0 (0)	2 (2.0)	0 (0)	0 (0)
11.0	Control	72	49 (68.1)	7 (9.7)	15 (20.8)	0 (0)	0 (0)	0 (0)	0 (0)	1 (1.4)	0 (0)	0 (0)
	0.2 Gy	144	93 (64.6)	7 (4.7)	30 (20.8)	10 (6.9)	2 (1.4)	0 (0)	0 (0)	2 (1.4)	0 (0)	0 (0)
21.6	Control	48	29 (60.4)	3 (6.3)	12 (25.0)	4 (8.3)	0 (0)	0 (0)	0 (0)	0 (0)	0 (0)	0 (0)
	0.2 Gy	127	71 (55.9)	19 (15.0)	21 (16.5)	9 (7.1)	2 (1.6)	3 (2.4)	0 (0)	0 (0)	0 (0)	2 (1.6)
34.6	Control	98	52 (53.1)	10 (10.2)	29 (29.6)	6 (6.1)	0 (0)	1 (1.0)	0 (0)	0 (0)	1 (1.0)	0 (0)
	0.2 Gy	183	81 (44.3)	35 (19.1)	49 (26.8)	15 (8.2)	1 (0.5)	0 (0)	0 (0)	1 (0.5)	0 (0)	1 (0.5)
192	Control	16	10 (62.5)	2 (12.5)	4 (25.0)	0 (0)	0 (0)	0 (0)	0 (0)	0 (0)	0 (0)	0 (0)
	0.2 Gy	48	18 (37.5)	9 (18.8)	14 (29.2)	2 (4.2)	0 (0)	2 (4.2)	1 (2.1)	1 (2.1)	0 (0)	1 (2.1)

## DISCUSSION

The accumulation of tritium contaminated water at the Fukushima Dai-ichi NPP raised concerns about HTO contaminated water [14, 15]. Clarifying the biological effects of low dose or low dose rate HTO exposures, as well as providing strong evidence for the effects of such exposures is necessary in order to make any decisions on how to deal with contaminated water. It is clear that ionizing radiation induces DNA damage, even when the doses are quite low, because many reports shows that radiation induced gamma-H2AX or 53BP1 foci can be detected even at several milli-Gray exposures [16–18]. On the other hand, cells have multiple DNA damage repair systems and DNA damage does not directly correlate with cell viability, especially in low dose ranges [19, 20]. Therefore, it is necessary to detect any genetic alterations or epigenetic changes which remain following DNA damage repair, in order to assess the stochastic effects of low dose and low dose rate radiation.

In the present study, we analyzed the induction of HPRT-deficient mutations by low dose and low dose rate HTO exposures by using a hyper-sensitive system to detect somatic mutations which was established in our laboratory [6]. At a total dose of 0.2 Gy, a significant decrease in induced mutant frequencies was observed at dose rates less than 11.0 mGy/day. The mutant frequency seen with dose rates of less than 11.0 mGy/day were quite similar to the level seen in non-exposed controls. In addition, we could not detect any differences in the mutation spectra between non-irradiated controls and irradiated populations exposed at dose rates of less than 11.0 mGy/day. The present observations suggest the possibility that there is a dose rate threshold for somatic mutation induction by HTO. Yamamoto *et al.* [8, 9] reported that the frequency of thymic lymphoma remained at nearly control levels when mice were orally administered HTO at a dose rate of less than 12 mGy/day throughout their lives [8, 9]. Since the present results are consistent with those of Yamamoto *et al.* [9], it is interesting to note that a dose-rate threshold for the stochastic effects generated by HTO may exist at approximately 12.0 mGy/day in both animal models and in cellular responses.

Mutation spectra analysis was performed to investigate the type of HPRT-deficient mutants induced by low dose rate HTO exposures. Based on the minimum number of DSBs required to form the particular mutant, we classified mutants into four categories: spontaneous-type mutants representing the loss of all four STS markers on the human X-chromosome; a one-DSB type representing a deletion on the long arm containing the *HPRT1* gene; a two-DSBs-type representing deletions on both, the long arm and short arm; and a translocation type representing the loss of a centromere. A major mutation type which was observed was a spontaneous-type caused by chromosome loss, regardless of whether the cells were irradiated or not. This observation might result from the instability of the human X-chromosome in rodent cells [11–13]. At higher dose rates of 34.6 mGy/day and 192 mGy/day, the percentage of radiation type events (one-DSB type, two-DSBs-type and translocation type) tended to increase in the irradiated group when compared to the non-irradiated group. In the present hyper-sensitive cell system, it was shown that a major spontaneous mutation event results from the instability of the human X-chromosome in rodent cells, and that radiation-induced mutation events are characterized by partial deletions of the human X-chromosome due to the induction of DSBs by radiation [7]. A similar spectrum was observed in both, HTO-exposed and non-exposed cells with a 0.2 Gy exposure and radiation type mutants increased only in cells which were exposed at higher dose rates. This finding suggests that the mode of mutation may shift from a spontaneous-type to a radiation type following an increase in the dose rate. This hypothesis is supported by the fact that the mutation spectra of the HTO-exposed group more closely resembled the control group at a dose rate of less than 11 mGy/day, when the mutant frequency was similar to spontaneous levels **(**Fig. 4**)**.

The probability of a DSB-induction which causes a deletion of the long arm of the human X-chromosome might be related to the appearance of a dose-rate threshold in the mutant frequency because an increase in the DXS1194 deletion was observed in mutants induced by an HTO dose rate between 21.6 mGy/day and 192 mGy/day in contrast to cells exposed to less than 11 mGy/day (Fig. 2C). The critical dose rate where the mutation spectrum changes is consistent with an inflection point in mutant frequency. Thus, a DSB which results in cells which have lost almost all of the long arm of the human X-chromosome may occur at higher dose rates, but not at dose-rates of less than 11 mGy/day. Russel and Hunsicker [21] analyzed historical data for radiation-induced mouse-specific locus mutations. They showed that only ‘large lesion’ (deletions on a chromosome) events increased at a dose-rate of > 0.8 R/min [21].

We would like to propose a possible mechanism that could result in more radiation-type deletions on the human X-chromosome at higher HTO dose-rates. First, we hypothesize that a hotspot for injuries from ionizing radiation might exist on the long arm of the human X-chromosome near the centromere. This idea is supported by the observations of Xiao and Natarajan [22] which described the existence of a fragile site near the centromere on the long arm of the X-chromosome (Xq21) in Chinese hamster cells [22]. Second, note that increasing dose rates lead to an increased number of DSBs in the nucleus, resulting in increased DNA damage on the long arm of the human X-chromosome per unit time. Third, it is likely that the quality of DSB repair might exhibit a higher fidelity when the damage per unit time is relatively small. Thus, at higher dose rates, a radiation type mutation can occur more efficiently due to substantial damage per unit time during the exposure period. It should be noted that the percentage of radiation type mutations in the HTO-exposed group approached control group values depending on the length of the culture period (Fig. 4). This phenomenon might occur because the possibility exists that human X-chromosomes are lost during the prolonged culture period used for low dose rate exposures. In other words, because human chromosomes transfected into rodent cells are selectively deleted [11–13], the proportion of DNA damage-induced mutation events on the human X-chromosome could be masked by the loss of the human X-chromosome during prolonged culture periods.

It remains to be understood why the induced mutant frequency and mutation spectrum clearly changed between 11 mGy/day and 21.6 mGy/day. Arcanjo *et al*. [23, 24] exposed zebrafish eggs at two HTO concentrations corresponding to dose-rates of 0.4 mGy/h and 4 mGy/h, and investigated the effect on the transcriptome [23, 24]. They reported that the genes involved in DNA repair such as *h2afx*, *bcl2l* and *xrcc1* were upregulated when eggs were irradiated at 4 mGy/h, whereas the genes involved in promoting apoptosis were upregulated when eggs were irradiated at 0.4 mGy/h. Their results suggest that there may be a critical dose rate where changes in cellular responses to radiation at the molecular level occur. The present study supports the existence of an inflection point in cellular responses to radiation which depends not only on the dose, but also on the dose rate. Further detailed studies will be designed to clarify the mechanisms involved.

Although our hypersensitive system can detect mutagenic effects at about a 50-fold higher frequency than conventional systems using the *HPRT1* gene, the higher frequency of spontaneous mutations observed, especially after long term exposures, remains to be studied. To obtain a more detailed analysis of the mutagenic effects of low dose or low dose rate radiation, it will be necessary to establish a novel system in which spontaneous mutations can be suppressed.

In summary, the present study shows that the mutation events generated by HTO are similar to those observed for spontaneous events at dose rates below 11 mGy/day. Our results suggest the existence of a dose rate threshold for mutagenic events generated by HTO because a transition in the composition of the mutation spectra can be seen at a dose-rate below 11 mGy/day. Improving the experimental system should help resolve biological events which occur in response to very low levels of HTO exposures.
